# Anti-inflammatory effects of indirubin derivatives on influenza A virus-infected human pulmonary microvascular endothelial cells

**DOI:** 10.1038/srep18941

**Published:** 2016-01-06

**Authors:** Hoi-Hin Kwok, Po-Ying Poon, Siu-Ping Fok, Patrick Ying-Kit Yue, Nai-Ki Mak, Michael Chi-Wai Chan, Joseph Sriyal Malik Peiris, Ricky Ngok-Shun Wong

**Affiliations:** 1 Dr. Gilbert Hung Ginseng Laboratory, Faculty of Science, Hong Kong Baptist University, Kowloon Tong, Hong Kong Special Administrative Region; 2 Department of Biology, Faculty of Science, Hong Kong Baptist University, Kowloon Tong, Hong Kong Special Administrative Region; 3 Centre of Influenza Research and School of Public Health, Li Ka Shing Faculty of Medicine, The University of Hong Kong, Hong Kong Special Administrative Region

## Abstract

Influenza A virus (IAV) poses global threats to human health. Acute respiratory distress syndrome and multi-organ dysfunction are major complications in patients with severe influenza infection. This may be explained by the recent studies which highlighted the role of the pulmonary endothelium as the center of innate immune cells recruitment and excessive pro-inflammatory cytokines production. In this report, we examined the potential immunomodulatory effects of two indirubin derivatives, indirubin-3′-(2,3-dihydroxypropyl)-oximether (E804) and indirubin-3′-oxime (E231), on IAV (H9N2) infected-human pulmonary microvascular endothelial cells (HPMECs). Infection of H9N2 on HPMECs induced a high level of chemokines and cytokines production including IP-10, RANTES, IL-6, IFN-β and IFN-γ1. Post-treatment of E804 or E231 could significantly suppress the production of these cytokines. H9N2 infection rapidly triggered the activation of innate immunity through phosphorylation of signaling molecules including mitogen-activated protein kinases (MAPKs) and signal transducer and activator of transcription (STAT) proteins. Using specific inhibitors or small-interfering RNA, we confirmed that indirubin derivatives can suppress H9N2-induced cytokines production through MAPKs and STAT3 signaling pathways. These results underscore the immunomodulatory effects of indirubin derivatives on pulmonary endothelium and its therapeutic potential on IAV-infection.

Influenza A viruses (IAV) cause seasonal epidemics and occasional global pandemics in human populations and resulted in a substantial number of deaths and economic burden^1^. IAV are single-stranded negative-sense RNA viruses that belong to the family Orthomyxoviridae. Their RNA genome is comprised of eight segments which encode for 11 viral proteins including the surface proteins hemagglutinin (HA) and neuraminidase (NA), matrix proteins M1 and M2, nonstructural proteins NS1 and NS2, and polymerase proteins PB1, PB2, PA, and PB1-F2^2^. The glycoproteins HA and NA play a determinative role in viral tropism as well as pathogenesis. For instance, seasonal H3N2 virus mainly bind onto the epithelium of the upper respiratory track, while highly pathogenic avian H5N1 attaches abundantly to the lower respiratory tract^3^.

Nevertheless, infection of the virus triggers an immediate innate immune response of the host cells in order to restrict the spread of the virus. The host pathogen recognition receptors (PRRs) play a vital role in recognizing pathogen-associated molecular patterns (PAMPs) from invading pathogens. Its activation initiates and orchestrates the innate immunity during an infection^4^. Transmembrane toll-like receptors (TLRs), such as TLR-3^5^/7^6^/8^7^/10^8^ and retinoic acid-inducible gene-I-like receptors (RLRs)^9^ can recognize influenza viral protein or viral RNA molecules. Recognition of IAV by the host cell activates several intracellular signaling pathways and results in the induction of gene expression for cytokine or chemokines^10^. These cytokines and chemokines are essential in cell-cell communication and recruitment of immune cells. Gene expression of cytokines is tightly regulated by a complex network of signaling pathway. Mitogen-activated protein kinases (MAPKs), including p38 MAPK (p38), c-Jun N-terminal kinase (JNK) and extracellular signal-regulated kinase (ERK), are the most extensively studied signaling pathway in the context of innate immunity^11^. Each MAPK has a distinct role in conveying the effects of PRRs activation. In general, JNK activation is pro-inflammatory^12^, while p38 and ERK play a role in both eliciting and turning-off inflammatory responses^13^,^14^,^15^. Binding of cytokines on their transmembrane receptor leads to activation of downstream signaling pathways, signal transducer and activator of transcription (STAT) proteins are the common signaling molecules which function as transcription factors for cytokines production^16^,^17^.

The epithelium of the human conducting airway^18^,^19^ and lung alveolus (Type 1 or 2 pneumocytes)^20^ serve as the primary target of IAV. However, infection of IAV induces the alveolus epithelial cells to produce cytokines that can further activate the endothelial cells on its basolateral side^21^. Recent studies on highly pathogenic avian influenza viruses like H5N1 subtype highlighted that lung endothelium are at the center of innate immune cells recruitment and excessive pro-inflammatory cytokine production during severe IAV infection^22^,^23^,^24^. Clinical presentation of severe IAV infection is characterized by multi-organ failure and systemic inflammatory response syndrome, also known as a “cytokine storm”^25^,^26^. Thus, immunomodulation of lung endothelium may serve as an attractive therapeutic strategy for the treatment of IAV infection^27^,^28^,^29^.

Currently, the primary means of prevention against influenza is annual vaccination. However, the availability of vaccine may be overwhelmed by the rapid spread of IAV^30^. Also, influenza targeting agents like Amantadine and Rimantadine (M2-ion channel inhibitors) or Oseltamivir and Zanamivir (NA inhibitors) may select for mutational escape and show widespread resistance^31^. In addition, use of antiviral agents alone may not be enough for IAV-infected patients with over-activated immune responses. Modulation of the host immune response has the potential advantage to overcome the above problems^32^.

The search for novel antiviral and immunomodulatory drugs against influenza concentrates not only on synthesis of new drugs, but also compounds isolated from natural sources^33^. Our previous study showed that ginsenosides derived from *Panax ginseng* have anti-inflammatory effects on IAV-infected endothelial cells^34^. Indirubin originates from the root of herbal plant *Isatis indigotica*, *Strobilanthes cusia* and *Polygonum tinctorium*, has been used to treat sore throat and flu in Chinese Medicine. Molecular studies have shown its potent inhibitory effects on various kinases, including mitogen-activated protein kinases (MAPKs)^35^,^36^ and Src kinases^37^. Since indirubin shows poor solubility and bioavailability, several derivatives have been chemically synthesized to improve its functional activities. It has been previously shown that indirubin and its derivatives are potent antiviral^38^,^39^ and anti-inflammatory^40^,^41^,^42^ compounds. Here we investigated the immunomodulatory effects of two indirubin derivatives, indirubin-3′-(2,3-dihydroxypropyl)-oximether (E804) and indirubin-3′-oxime (E231) on influenza A virus (H9N2) infected-human pulmonary microvascular endothelial cells (HPMECs).

## Material and Methods

### Chemicals

Indirubin-3′-oxime (E231) was obtained from Sigma-Aldrich (St. Louis, MO, USA). Indirubin-3′-(2,3-dihydroxypropyl)-oximether (E804) was purchased from Calbiochem (La Jolla, CA, USA). The following antibodies, including anti-phospho p38 (Thr^180^/Tyr^182^), anti-p38, anti-phospho JNK (Thr^183^/Tyr^185^), anti-JNK, anti-phospho ERK (Thr^202^/Tyr^204^), anti-ERK, anti-STAT3, and anti-phospho STAT3 (Tyr^705^) were obtained from Cell Signaling Technology (Beverly, MA, USA). Anti-GAPDH and anti-lamin A/C were purchased from Santa Cruz Biotechnology (Santa Cruz, CA, USA). Secondary antibody was purchased from Invitrogen (Carlsbad, CA, USA). Endothelial cell growth supplement (ECGS) was purchased from Millipore (Billcerica, MA, USA). Penicillin and streptomycin mixture (PS) and fetal bovine serum (FBS) were purchased from Invitrogen. Other chemicals not specified are obtained from Affymetrix USB Chemical (Cleveland, OH, USA).

### Influenza A virus

Influenza A/H9N2 virus (A/Quail/HongKong/G1/97) was used in this study. Viruses were cultured in MDCK cells (ATCC, Manassas, VA, USA) before the infection experiments. Virus infectivity was assessed by plaque forming assay in MDCK cells. Isolation of influenza A virus (IAV) subtype A/Quail/Hong Kong/G1/97 (H9N2) was described elsewhere^43^.

### Cell cultures and influenza A virus infection

Human pulmonary microvascular endothelial cells (HPMECs) were obtained from ScienCell (San Diego, CA, USA). Cells were cultured in M199 medium supplemented with heparin (90 mg/L, w/v), heat-inactivated FBS (20%, v/v), ECGS (20 μg/ml) and PS (1%, v/v) and kept at incubator maintained in 37 °C and 5% CO_2_. HPMECs were passaged and assayed within the passage 3–11 to ensure genetic stability. Cells were seeded on gelatin (0.1%)-coated cell culture plate. After a PBS wash, HPMECs were inoculated with IAV using a multiplicity of infection (MOI) of 2 at 37 °C. After 1 h of infection, cells were washed with PBS and then treated with or without indirubin derivatives. Mock-infected cells were used as negative control.

### Quantitative analysis of cytokines by enzyme-linked immunosorbent assays (ELISA)

After treating with indirubin derivatives for 24 h, culture supernatant was harvested. The levels of cytokines (IP-10, RANTES, IL-6, IL-1β, MCP-1, TNF-α,) were quantified by sandwich ELISA according to the manufacturer’s protocol (R&D Systems, Minneappolis, MN, USA).

### Cell viability assays

HPMECs (1 × 10^4^ cells/well) were seeded onto 96-well plates. After treatment with various concentrations of indirubin derivatives for the indicated time, cell viability was determined by MTT assay^44^. The absorbance at wavelength of 540 nm was measured by the ELx800 Absorbance Reader (BioTek, Winooski, VT, USA). Percentage of cell viability was determined by the following formula: Absorbance _treatment group_ – Absorbance _blank_)/(Absorbance _vehicle control group_ – Absorbance _blank_).

### Real-time reverse transcription-polymerase chain reaction (RT-PCR) for mRNA quantitation

After treatment, total RNA in HPMECs was extracted by TRIzol (Invitrogen) following the manufacturer’s instruction. Complementary DNA was synthesized from DNase-treated total RNA using Superscript II first-strand synthesis system (Invitrogen). The relative expression of target gene was quantified by real-time RT-PCR using KAPA SYBR Fast ABI prism qPCR Kit (KAPA Biosystems, Woburn, MA, USA) and detected by a StepOnePlus real-time PCR system (Applied Biosystems, Foster City, CA, USA). The relative expression of target gene was normalized by the level of glyceraldehyde-3-phosphate dehydrogenase (GAPDH), and then calculated by the comparative Ct method.

### Plaque assay

The virus titers were determined by standard plaque assay on Madin-Darby canine kidney (MDCK) cells. In brief, MDCK cells were grown in MEM and seeded onto 6-well plates. Diluted cell culture medium from influenza virus-infected HPMECs were added to the confluent MDCK cells monolayers for 1h. Then, the inoculum was removed, and a mixture of agarose (2%, w/v) containing L-(tosylamido-2-phenyl) ethyl chloromethyl ketone (TPCK) (1 μg/ml) was added onto the MDCK cells monolayers. After 72 h of incubation, the plate was fixed by formaldehyde (4%, v/v) overnight and then the agarose was discarded. The plaques were counted after staining with crystal violet (0.2%, w/v).

### Subcellular fractionation and Western blot analysis

Cytoplasmic and nuclear extract were prepared by NE-PER Nuclear and Cytoplasmic Extraction Reagents (Thermo Scientific, Rockford, IL, USA) according to the manufacturer’s protocol. For extraction of whole-cell lysate, cells were lysed by CytoBuster^TM^ Protein Extraction Reagent (Novagen, Madison, WI, USA) containing protease (0.5%, v/v) and phosphatase inhibitor cocktails (0.5%, v/v) (Calbiochem, San Diego, CA, USA). The cell lysate was collected after centrifugation. Protein concentration of the sample was determined by the detergent-compatible protein assay (Bio-Rad, Hercules, CA, USA). Equal amounts of protein were loaded and separated by 10% SDS-PAGE followed by electroblotting onto nitrocellulose membrane. The membrane was soaked in blocking buffer (1% non-fat milk in TBS-T, w/v) and then incubated with specific primary antibodies overnight at 4 °C and secondary antibody for 1 h at room temperature. Immunoreactive bands were visualized using SuperSignal West Pico Kit (Thermo Scientific).

### *In vitro* mitogen-activated protein kinases assay

To detect the activity of individual MAPKs after treatment with IAV and indirubin derivatives, the non-radioactive *In vitro* protein kinase assay kit from Cell Signaling Technology was used. In brief, the Sepharose bead-immobilized antibody was used to immunoprecipitate active MAPKs from an equal amount of total cell lysate (200 μg) overnight. The immunoprecipitate was washed twice with cell lysis buffer and kinase reaction buffer. The immunoprecipitate were then incubated with indirubin derivatives E804 or E231 (1 μM) for 3 min before addition of ATP. Subsequently, kinase reactions using corresponding protein substrate were performed at 37 °C for 30 min. The kinase reaction was stopped with SDS loading buffer. Phosphorylation of protein substrate was detected by immunoblotting with specific antibody.

### Immunofluorescence microscopy

HPMECs at a density 1 × 10^4^ were seeded onto a glass coverslip in a 24-well plate. After treatment for the indicated time, cells were fixed with 4% paraformaldehyde for 15 min at room temperature. Cells were incubated with primary antibody (1: 200 dilution rabbit anti-phospho-STAT3 (Tyr^705^) antibody) overnight at 4 °C. The coverslip was washed and then incubated with FITC-conjugated goat anti-rabbit secondary antibody (1: 250 dilution) (Invitrogen) for 2 h at room temperature. Nuclei were visualized by staining with DAPI (0.5 μg/ml). The coverslip was washed and mounted on a slide using DAKO fluorescence mounting medium (Carpinteria, CA, USA). Fluorescence image was captured by the Olympus Fluoview FV1000 confocal laser-scanning microscope (Tokyo, Japan).

### Small interfering RNA (siRNA) transfection

Transfection of siRNA was performed using Lipofectamine RNAiMAX (Invitrogen). Non-targeting-siRNA (50 nM) was used in parallel with STAT3-specific siRNA (50 nM) (Ambion, Austin, TX, USA). Cells plated at 80% confluence were transfected in Opti-MEM medium (Gibco BRL, Grand Island, NY, USA) for 24 h. After transfection, cells were rinsed with Opti-MEM prior to further treatment.

### Statistical analysis

All results were expressed as mean ± standard derivation (S.D.) of at least 3 independent experiments. Statistical significance between groups was determined by one-way ANOVA with Tukey’s post hoc test. *p* < 0.05 was considered to be statistically significant.

## Results

### Influenza A virus H9N2 is a potent inducer of cytokines production in pulmonary endothelial cells

**Figure 1:**
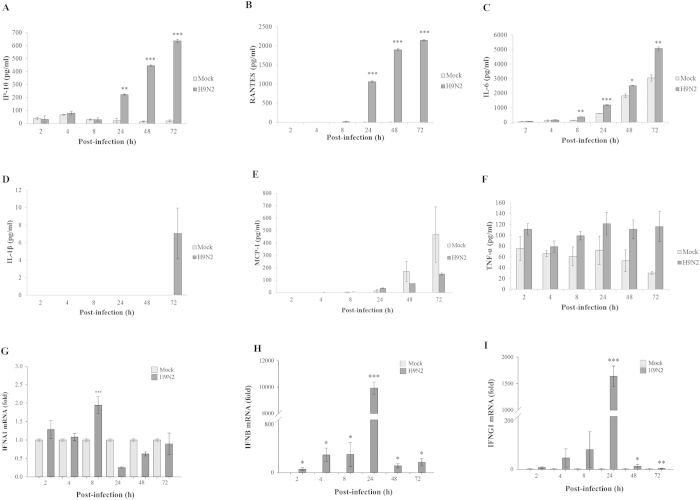
Infection of H9N2 on human pulmonary endothelial cells strongly induces expression of multiple cytokines. HPMECs were infected with H9N2 at 2 MOI. Cell culture supernatant or total RNA were harvested at the indicated time point. The levels of (**A**) IP-10, (**B**) RANTES, (**C**) IL-6, (**D**) IL-1β, (**E**) MCP-1, and (**F**) TNF-α were measured by ELISA. The mRNA levels of (**G**) IFN-α1, (**H**) IFN-β and (**I**) IFN-γ1 were quantified by real-time RT-PCR. The values are presented as mean ± S.D. from three independent experiments. *p < 0.05, **p < 0.01, ***p < 0.001 vs mock-infected cells.

Recent studies suggested that lung endothelium is the central regulator of cytokine amplification during influenza A virus infection, while dysregulation of cytokines production may result in systemic inflammation^22^. In this study, we found that the infection of influenza A virus subtype A/Quali/Hong Kong/G1/97 (H9N2) on HPMECs induced excessive production of various pro-inflammatory cytokines and chemokines, including IP-10 (Fig. 1A), RANTES (Fig. 1B) and IL-6 (Fig. 1C) in a time-related manner. IL-6 was first induced at 8 h post-infection (h.p.i.), IP-10 and RANTES were induced after 24 h.p.i. However, H9N2 did not significantly increase IL-1β (Fig. 1D), MCP-1 (Fig. 1E) and TNF-α (Fig. 1F) in HPMECs. Meanwhile, H9N2-infection in HPMECs strongly induced IFN-β (Fig. 1H) and IFN-γ1 mRNAs (Fig. 1I), but only transient IFN-α1 mRNA (Fig. 1G) induction at 8 h.p.i. Both IFN-β and IFN-γ1 mRNAs expressions peak at 24 h.p.i.

### Indirubin derivatives inhibit H9N2-induced cytokines expression in pulmonary endothelial cells

**Figure 2:**
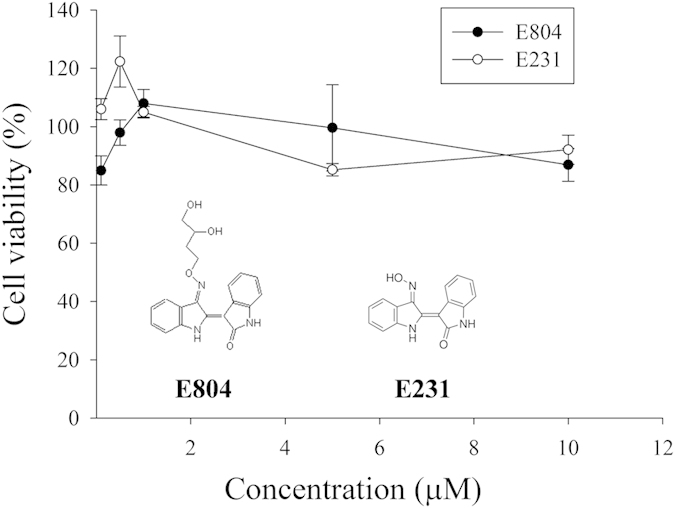
The cytotoxicities of indirubin derivatives E804 and E231. HPMECs were incubated with E804 or E231 (0.1, 0.5, 1, 5 and 10 μM) for 24 h. Cell viability was determined by MTT assay. The values are presented as mean ± S.D. from three independent experiments.

**Figure 3:**
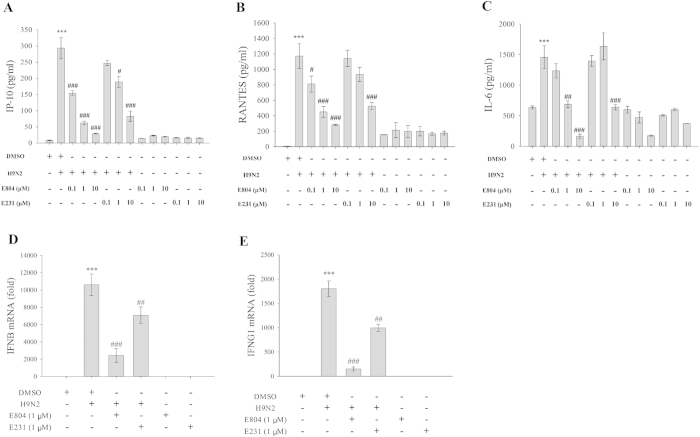
Indirubin derivatives E804 and E231 suppress H9N2-induced cytokines production in HPMECs in a concentration-related manner. HPMECs were infected with H9N2 at 2 MOI followed by treatment with indirubin derivatives E804 or E231 (0.1, 1, 10 μM) for another 24 h. Cell culture supernatant and total RNA were harvested and the levels of (**A**) IP-10, (**B**) RANTES and (**C**) IL-6 were measured by ELISA. The mRNA levels of (**D**) IFN-β and (**E**) IFN-γ1 were quantified by real-time RT-PCR. The values are presented as mean ± S.D. from three independent experiments. **p < 0.01, ***p < 0.001 vs mock-infected cells. ^#^*p* < 0.05, ^##^*p* < 0.01, ^###^*p* < 0.001 vs H9N2-infected cells.

**Figure 4:**
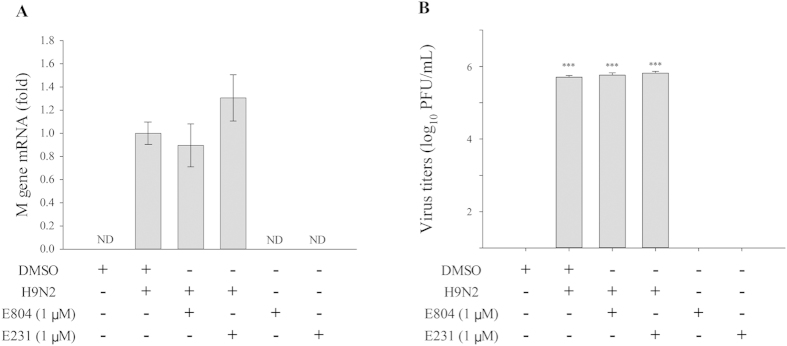
Indirubin derivatives E804 and E231 do not affect H9N2 replication in human pulmonary endothelial cells. HPMECs were infected with H9N2 at 2 MOI followed by treatment with indirubin derivatives E804 or E231 (1 μM) for another 24 h. (**A**) The mRNA level of influenza M1 gene was quantified by real-time RT-PCR. (**B**) The viral titers were quantified by standard plaque formation assay in MDCK cells. The values are presented as mean ± S.D. from three independent experiments. ***p < 0.001 vs mock-infected cells. ND = Not Detected.

To examine the immunomodulatory effects of indirubin derivatives, HPMECs were infected with H9N2 for 1 h followed by incubation with indirubin derivatives E804 or E231 for another 24 h. We have tested the cytotoxicities of indirubin derivatives in HPMECs prior to the ELISA. As shown in Fig. 2, no significant cytotoxicity was observed at or below 10 μM in HPMCEs. Next, indirubin derivatives were found to suppress H9N2-induced IP-10 (Fig. 3A), RANTES (Fig. 3B) and IL-6 (Fig. 3C) expression in a concentration-related manner. E804 significantly inhibited cytokines expression at 1 μM, a similar inhibitory effect was observed when a higher concentration of E231 (10 μM) was used. Both indirubin derivatives slightly induced and inhibited the basal level of RANTES and IL-6 respectively. It may be due to the regulatory effects of indirubins on innate immunity^45^. For the IFN-β (Fig. 3D) and IFN-γ1, (Fig. 3E) both E804 and E231 (1 μM) could significantly reduce H9N2-induced expression. On the other hand, to examine the antiviral property of indirubin derivatives, we measured the viral matrix 1 (M1) gene expression in HPMECs. The RT-PCR result showed that H9N2 could efficiently replicate and transcribe viral protein in HPMECs, but neither indirubin derivatives E804 nor E231 could reduce H9N2 M1 gene expression (Fig. 4A). The plaque formation assay further confirmed efficient virus replication in HPMECs (Fig. 4B). However, both indirubin derivatives cannot reduce the virus titer in H9N2-infected HPMECs.

### H9N2-induced IP-10, RANTES and IL-6 expressions are mediated through MAPKs

**Figure 5:**
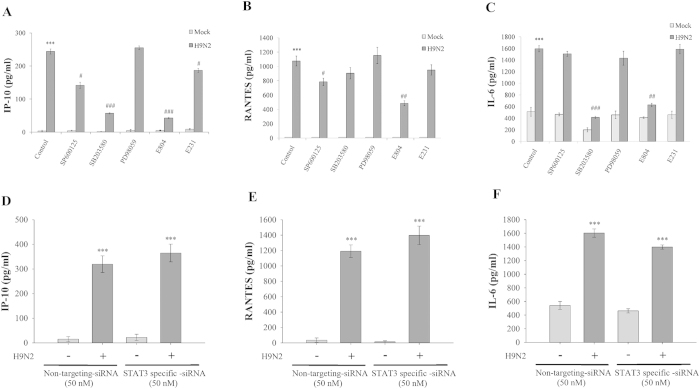
H9N2-induced IP-10, RANTES and IL-6 productions in human pulmonary endothelial cells are mediated through MAPKs. (**A–C**) HPMECs were infected with H9N2 at 2 MOI followed by treatment with either SP600125 (JNK inhibitors) (10 μM), SB203580 (p38 inhibitor) (10 μM), PD98059 (ERK inhibitor) (10 μM), indirubin derivatives E804 (1 μM) or E231 (1 μM) for another 24 h. (**D–F**) HPMECs transfected with non-targeting siRNA (50 nM) or STAT3 specific siRNA (50 nM) were infected with H9N2 at 2 MOI for 1 h, and further incubated for 24 h.p.i. Cell culture supernatant was harvested and the levels of IP-10, RANTES and IL-6 were measured by ELISA. The values are presented as mean ± S.D. from three independent experiments. ***p < 0.001 vs mock-infected cells. ^#^*p* < 0.05, ^##^*p* < 0.01, ^###^*p* < 0.001 vs H9N2-infected cells.

To investigate the signaling pathways that mediate the induction of cytokines production during H9N2 infection in HPMECs, cells were treated with MAPKs specific chemical inhibitors after H9N2 infection, or transfected with STAT3-specific siRNA before H9N2 infection. The ELISA results showed that JNK inhibitor SP600125 partially inhibited H9N2-induced IP-10 (Fig. 5A) and RANTES (Fig. 5B) production. Also, the p38 inhibitor SB203580 could significantly suppress the production of IL-6 (Fig. 5C) and showed partial inhibition on IP-10 (Fig. 5A) expression induced by H9N2. To confirm the suppressive effects of different MAPKs inhibitors on cytokines are not due to impaired viral replication, we introduced viral RNA into HPMECs as a non-dynamic viral stimulus. HPMECs were transfected with viral RNA or cellular RNA in the presence or absence of different MAPKs inhibitors (Supplementary figure 1)^46^. Similar to the results of direct virus infection, viral RNA stimulated cytokines expressions, and the inhibitory effects of MAPKs inhibitors were very similar to the experiments in direct infection. This confirmed that the suppression effects of MAPKs inhibitors on cytokines expression were not due to their potential effects on viral load. As a result, the anti-inflammatory effects of indirubin derivatives are mainly due to its inhibitory effects on different kinases.

**Figure 6:**
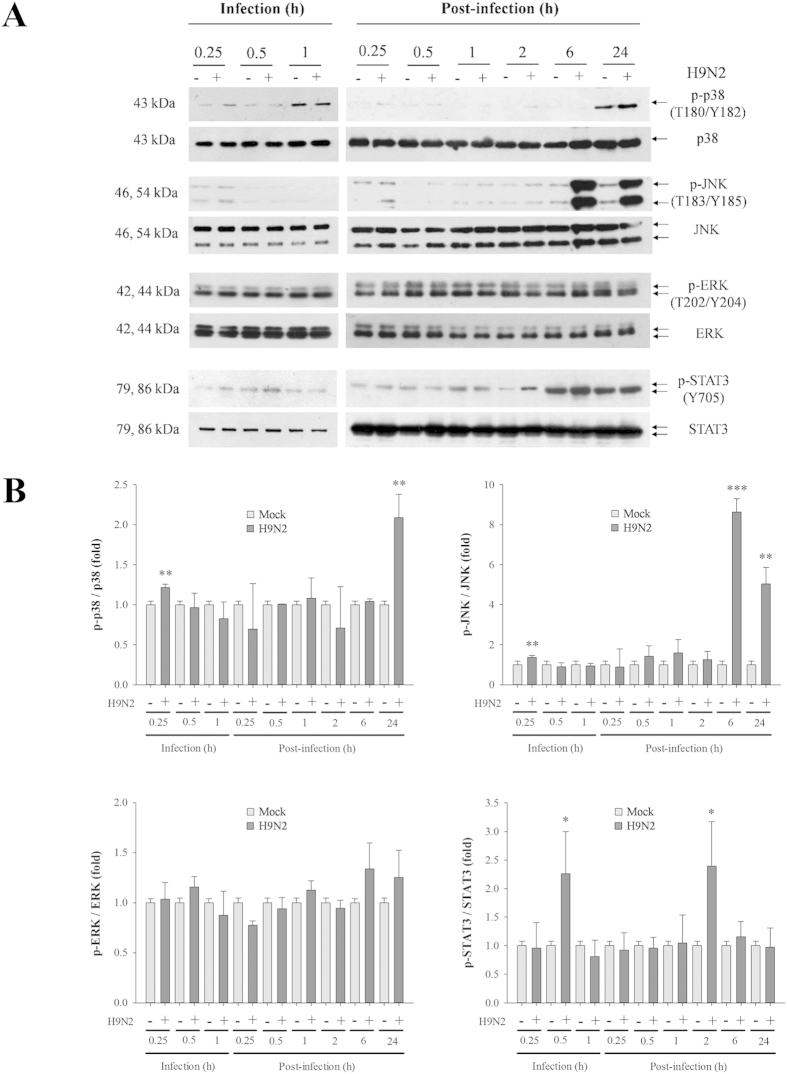
Infection of H9N2 on human pulmonary endothelial cells induces rapid phosphorylation of p38, JNK, and STAT3. HPMECs were infected with H9N2 at 2 MOI. Total cell lysates were harvested at the indicated time (0.25, 0.5 and 1 h after addition of virus, and 0.25, 0.5, 1, 6 and 24 h.p.i.). Phosphorylation and total p38, JNK, ERK and STAT3 were detected by Western blot analysis with specific antibodies. (**A**) Representative image from three independent experiments. (**B**) Quantitative analysis of Western blotting. Values are presented as fold-change of phosphorylated p38, JNK, ERK or STAT3 normalized to the corresponding total form and then compared with mock-infected cells. The values are presented as mean ± S.D. from three independent experiments. *p < 0.05, **p < 0.01, ***p < 0.001 vs mock-infected cells.

In contrast, STAT3-specific siRNA had no effects on IP-10, RANTES and IL-6 production induced by H9N2 infection in HPMECs (Fig. 5D–F). Time-course experiments showed that H9N2 rapidly induced p38 and JNK phosphorylation in 15 min after addition of the virus, then a second wave of p38 and JNK phosphorylation were induced at 24 h.p.i. and 6 h.p.i., respectively (Fig. 6A,B). No activation of ERK was found after H9N2 infection. Similar to the early phosphorylation of stress-related MAPKs, STAT3 was phosphorylated early in 30 min after addition of the virus. However, the second wave of STAT3 phosphorylation was found at 2 h.p.i. Taken together, these results suggested that H9N2 infection on HPMECs could activate p38, JNK and STAT3 signaling pathways rapidly, and the expression of cytokines including IP-10, RANTES and IL-6 were mainly due to the activation of p38 and JNK.

### Indirubin derivatives suppress H9N2-induced cytokines expression through direct inhibition of p38 and JNK activity

**Figure 7:**
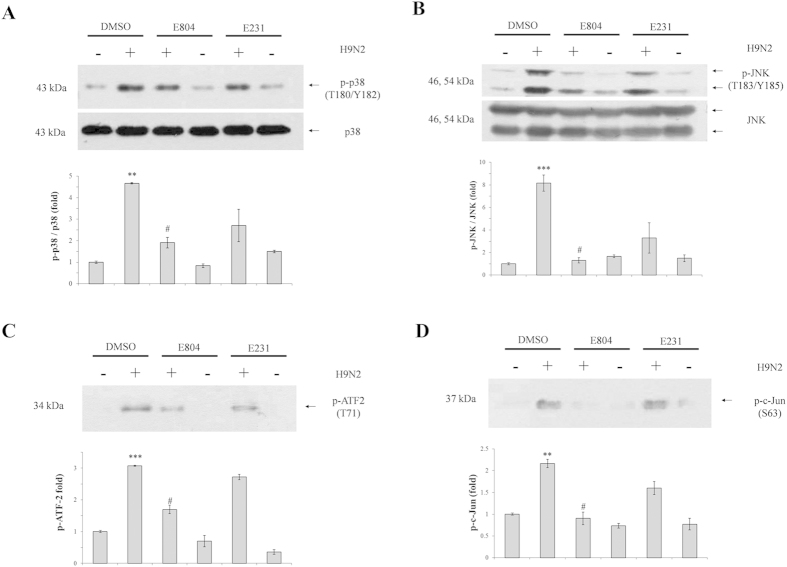
Indirubin derivatives E804 and E231 inhibit H9N2-induced p38 and JNK signaling pathways in human pulmonary endothelial cells. (**A,B**) HPMECs were infected with H9N2 at 2 MOI followed by treatment with indirubin derivatives E804 or E231 (1 μM). Total cell lysates were harvested at 24 h.p.i. (for p-p38) or 6 h.p.i. (for p-JNK). Phosphorylation and total p38 and JNK were detected by Western blot analysis with specific antibodies. Representative image from three independent experiments (Upper panel). Quantitative analysis of Western blotting. Values are presented as fold-change of phosphorylated p38 or JNK normalized to corresponding total form, and then compared with mock-infected cells (Lower panel). (**C,D**) E804 directly inhibits p38 and JNK kinases activity *in vitro*. HPMECs were infected with H9N2 at 2 MOI. Cell lysates were harvested at 24 h.p.i. (for p-p38) or 6 h.p.i. (for p-JNK). *In vitro* kinase assay of p38 and JNK was then performed as described in materials and methods. Representative image from three independent experiments (Upper panel). Quantitative analysis of Western blotting. Values are presented as fold-change of phosphorylated ATF2 or c-Jun compared with mock-infected cells (Lower panel). The values are presented as mean ± S.D. of three independent experiments. **p < 0.01, ***p < 0.001 vs mock-infected cells, ^#^*p* < 0.05 vs H9N2-infected cells.

To study the underlying mechanism of the anti-inflammatory effects of indirubin derivatives, HPMECs were treated with indirubin derivatives E804 or E231 after H9N2 infection. As shown in Fig. 7, E804 can significantly reduce H9N2-induced phosphorylation of p38 (Fig. 7A) and JNK (Fig. 7B) at 24 and 6 h.p.i., respectively, and E804 demonstrated a more potent effect than E231. It has been suggested that indirubin and its derivatives are potent inhibitors of various kinases, including MAPKs. *In vitro* kinase assay on p38 and JNK showed that E804 but not E231 inhibited p38 and JNK kinases activity. This action was reflected by the reduced phosphorylation of their direct downstream substrates ATF2 (Fig. 7C) and c-Jun (Fig. 7D) respectively.

### Indirubin derivatives prevent H9N2-induced IFN-β expression through inhibition of STAT3 phosphorylation and nuclear translocation

**Figure 8:**
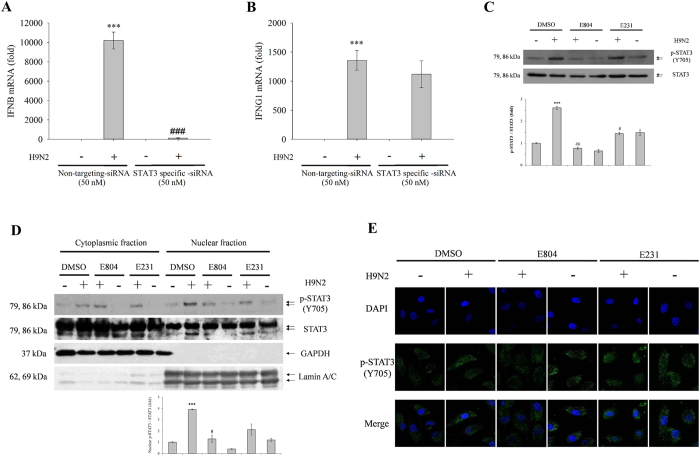
Indirubin derivatives suppress H9N2-induced IFN-β mRNA expression through inhibiting STAT3 signaling pathway. (**A,B**) HPMECs transfected with non-targeting siRNA (50 nM) or STAT3 specific siRNA (50 nM) were infected with H9N2 at 2 MOI for 1 h, and further incubated for 24 h.p.i. The mRNA levels of IFN-β and IFN-γ1 were quantified by real-time RT-PCR. (**C**) HPMECs were infected with H9N2 at 2 MOI followed by treatment with indirubin derivatives E804 or E231 (1 μM). Total cell lysates were harvested at 2 h.p.i. Phosphorylation and total STAT3 were detected by Western blot analysis with specific antibodies. (**D**) Subcellular fractionation was performed 2 h post infection. Western blot analysis was performed to determine the translocation of STAT3 from cytoplasm to nucleus. The GAPDH and Lamin A/C denoting the cytoplasmic and nuclear fractions respectively. Representative images from three independent experiments (Upper panel). Band intensities were determined by quantitative densitometry. Values are presented as fold-change of phosphorylated STAT3 normalized to total STAT3, and then compared with mock-infected cells (Lower panel). The values are presented as mean ± S.D. from three independent experiments. ***p < 0.001 vs mock-infected cells, ^###^*p* < 0.001 vs H9N2-infected cells. (**E**) Immunofluorescent staining of HPMECs with anti-phosphorylated STAT3 (Y705) antibody (Green). DAPI staining (Blue) was used to show the nucleus.

Though we found no relationship between STAT3 and H9N2-induced IP-10, RANTES and IL-6 expressions (Fig. 5D–F), STAT signaling pathway is indispensable for the induction of interferons. We showed that knockdown of STAT3 strongly inhibited H9N2-induced IFN-β (Fig. 8A) but not IFN-γ1 (Fig. 8B) mRNA expression. To elucidate the inhibitory effects of E804 on STAT3, Western blot analysis was performed. Treatment with E804 or E231 inhibited H9N2-induced STAT3 tyrosine phosphorylation (Fig. 8C). Upon activation, STAT3 forms homo- or heterodimers that translocate to the nucleus. HPMECs fractionation of nucleus and cytoplasm was obtained by means of subcellular fractionation followed by Western blot analysis. The results showed that H9N2 infection increased phosphorylated STAT3 in the nuclear fraction, while treatment with E804 significantly reduces the nuclear translocation (Fig. 8D). Similar to the result of Western blot analysis, the confocal image also showed that increased fluorescence signal was found in the nucleus after H9N2 infection in HPMECs (Fig. 8E). Treatment with E804 reduced STAT3 fluorescence signal in the nucleus.

## Discussion

The emergence of IAV poses a serious global threat to human health. Besides regular epidemic outbreaks, severe pandemics like the 1918 Spanish flu and the more recent 2009 swine flu had caused enormous social and economic burden. Current treatment of IAV infection by M2-ion channel inhibitors or NA inhibitors emerged high frequency of resistance, and the efficacy and effectiveness of these antiviral drugs are limited by disappointing success rate^1^, so alternative or complementary therapies that modulate the signaling pathways utilized by IAV came into focus. In this report, we demonstrated that indirubin derivatives, particularly E804 is a potent immunomodulatory compound for IAV-infection *in vitro* by inhibiting intracellular signaling pathways in pulmonary endothelial cells.

During the early stage of IAV infection, innate immune cells are recruited to the site of infection and are associated with an overwhelming production of pro-inflammatory cytokines and chemokines. Endothelial cells in the pulmonary vasculature form a barrier between the blood and interstitium. This strategic position suggests that pulmonary endothelial cells are prone to be affected by the cytokines and viral particles released from the IAV-infected epithelial cells. A recent study by Teijaro *et al*.> identified endothelial cells as the central orchestrator which contribute to the aberrant pro-inflammatory cytokine and chemokine production during early IAV infections^22^. Concomitant with our *in vitro* data, we showed that H9N2 virus can efficiently infect HPMECs (Fig. 4A,B) and induce a significant amount of IP-10, RANTEs, IL-6, IFN-β and IFN-γ1. IP-10 and RANTES are the chemoattractants for leukocytes including T cells, NK cells, and granulocytes, while production of IL-6 by endothelial cells initiates infiltration of neutrophils in the early phase of infection^47^. These cytokines have been found histopathologically in the lungs (including epithelial and endothelial cells) of H5N1 infected patients, who showed acute respiratory distress syndrome (ARDS)^48^, and ARDS can be characterized by progressive pulmonary endothelial damage. It has been suggested that treatment with antibodies against IP-10 in H1N1 infected mice can improve the survival rate and reduce acute lung injury^49^. Furthermore, suppression of early innate cytokine and chemokine production in the pulmonary endothelium can significantly improve survival of mice infected with lethal H1N1 Swine IAV^22^. These studies suggested that inhibition of cytokines production of the pulmonary endothelium is an attractive therapeutic strategy against IAV-induced cytokine storm.

Since severe infection of the influenza virus triggers the activation of the innate immune response and sometimes results in the induction to a cytokine storm. In this study, we demonstrated the immunomodulatory effects of indirubin derivatives, particularly E804 on IAV-infected pulmonary endothelial cells. Over the past two decades, many studies have identified that indirubin derivatives are potent inhibitors of various kinases, including MAPKs^35^,^36^, Src kinase^37^, glycogen synthase kinase-3β (GSK-3β)^50^ and cyclin-dependent kinases (CDKs)^51^. Based on these findings, potential functions of indirubin derivatives have been proposed, including anti-inflammation^35^,^40^,^41^,^42^, anti-leukemia^36^, antiviral^38^,^39^ and angiosuppression^52^,^53^. Crystal structure analysis revealed that indirubin can form three hydrogen bonds with the ATP-binding pocket of CDKs, thereby competitively inhibiting ATP binding in the catalytic domain of CDKs^36^. The results from our *in vitro* kinase assay also demonstrated that E804 is a potent inhibitor of p38 and JNK (Fig. 7C,D). The cytokine ELISA data also suggest that H9N2-induced IP-10 expression of HPMECs was dependent on p38 and JNK activation, while RANTES and IL-6 were controlled by JNK and p38, respectively (Fig. 5A–C). However, the Western blot analysis showed that E804 could also inhibit phosphorylation of p38 (Fig. 7A) and JNK (Fig. 7B), which mean upstream kinases of p38 and JNK may also be inhibited by E804. MAPKs are important mediators of influenza-induced cytokine expression. In fact, p38 has been shown to regulate the stability of IL-6 mRNA^54^. Meanwhile, another study also indicated the critical function of p38 on IP-10 during viral infection^55^. Furthermore, inhibition of p38 by specific inhibitor SB202190 *in vivo* can greatly diminish H5N1 lethal infection^28^. However, the role of JNK in IAV infection has not been fully examined. Nacken *et al*.> elucidated that influenza viral RNA induces JNK phosphorylation in an RIG-I dependent manner, but the NS1 of IAV has also an intrinsic JNK activating property^56^. Taken together, the potent inhibitory effect of p38 and JNK signaling pathways by E804 strongly correlates with its anti-inflammatory function.

IFNs are pro-inflammatory cytokines crucial for antiviral responses to IAV infection. STAT1 and STAT2 are predominant and essential transcription factors of type I and II interferons signaling pathway, but the role of STAT3 activation after IFN binding remains controversial. Undeniably, STAT3 is indispensable for downstream signaling pathway of many other cytokines like IL-6, VEGF or EGF^57^. Our data showed that H9N2 infection on HPMECs can significantly induce IFN-β and IFN-γ1 expression. Interestingly, STAT3-specific siRNA has no effect on H9N2-induced IL-6 (Fig. 5F) and IFN-γ1 (Fig. 8B), but strongly inhibit IFN-β expression level, indicating the involvement of STAT3 in IFN-β induction. The Western blot data showed that IAV-infection could activate STAT3 in early stage (15 min after addition of virus) followed by another activation at 2 h.p.i. (Fig. 6). H9N2 was found to upregulate TLR-8 and MyD88, which is critical to the induction of IFN-β^58^. In line with this finding, the early activation of STAT3 may function together with the downstream signaling molecules of TLR-8, possibly the interferon regulatory factors (IRFs), to induce rapid expression of IFN-β mRNA at 2 h.p.i. (Fig. 1H). And then the autocrine effect of IFN-β induces a further amplification of IFN-β expression and result in a cytokine storm. A previous study suggested that E804 could inhibit STAT3 dimerization and subsequent DNA binding^37^. Our translocation experiments clearly indicated that E804 could inhibit STAT3 phosphorylation and nuclear translocation. Since the induction of many pro-inflammatory cytokines requires type I interferon signaling, inhibition of IFN-β production by E804 may blunt the early induction of these cytokines. Though IFN-β is a well-known antiviral cytokine, it is also involved in the pathogenesis of influenza infection. *In vivo* studies focusing on the S1P_1_ receptor and p38 pathway also suggested that, even the IAV-induced IFNs were suppressed by an S1P_1_ receptor agonist^22^ or p38 inhibitor^28^, the survival rate of the infected mice could still be significantly improved if those strongly induced cytokines were suppressed. In many studies, increased viral load were concomitant of reduced interferons expressions. However, in the present study, viral titer did not further increased in indirubin derivatives treated cells (Fig. 4B) even IFNβ and IFN-γ1 were suppressed, the results indicated that suppression of cytokines produced by the infected pulmonary endothelium could reduce IAV pathogenicity independent of the viral clearance. In fact, many kinases including CDK^39^ and MAPKs, which are suggested being involved in the influenza replication process are also the target of indirubin derivatives, this might explain the partial antiviral effect of indirubin derivatives in this model.

## Conclusions

**Figure 9:**
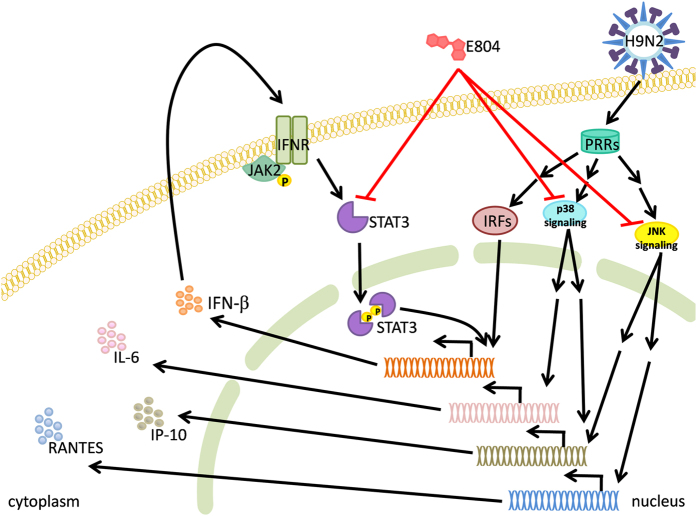
The schematic figure of immunomodulatory effects of indirubin derivatives against H9N2-infected HPMCEs. Infection of H9N2 on HPMECs induced rapid phosphorylation of p38, JNK and STAT3. H9N2-induced expression of IP-10, RANTEs and IL-6 were mediated through JNK and p38 signaling pathways. Meanwhile the phosphorylation and nuclear translocation of STAT3 leaded to induction of IFN-β. Indirubin derivatives particularly E804 is a potent inhibitor of p38 and JNK signaling pathways. E804 could also reduce the phosphorylation and nuclear translocation of STAT3. By inhibition of these signaling pathways, E804 could significantly suppress H9N2-induced cytokine burst in HPMECs.

Combination therapies coupling with antiviral and immunomodulatory drugs have been investigated intensively^29^. The encouraging results from *in vitro* and pre-clinical studies have led to an increased interest on this topic. However, to achieve the best clinical outcome, antiviral and immunomodulatory drugs should be administrated at the appropriate time during an infection. Further understanding of the immune dynamic could allow us to design an optimum therapy strategy. In this report, we demonstrated for the first time, the potent immunomodulatory effects of indirubin derivatives on pulmonary endothelial cells and their therapeutic potential for IAV-infection (Fig. 9). As a result, the combinational effects of indirubin derivatives and antiviral drug in animal model warrant further investigation.

## Additional Information

**How to cite this article**: Kwok, H.-H. *et al*.> Anti-inflammatory effects of indirubin derivatives on influenza A virus-infected human pulmonary microvascular endothelial cells. *Sci. Rep.* **6**, 18941; doi: 10.1038/srep18941 (2016).

## Supplementary Material

Supplementary Information
