# Supplementary material for: Anti-inflammatory effects of indirubin derivatives on influenza A virus-infected human pulmonary microvascular endothelial cells
Source: Sci Rep. 2016 Jan 6;6:18941. doi: 10.1038/srep18941 (PMC4702174; doi:10.1038/srep18941)
Supplement: Supplementary Information [file srep18941-s1.pdf]

Anti-inflammatory effects of indirubin derivatives on influenza A virus-infected human pulmonary microvascular endothelial cells

Hoi-Hin Kwok[1,2,#], Po-Ying Poon[1,2,#], Siu-Ping Fok[1,2], Patrick Ying-Kit Yue[1,2], Nai-Ki Mak[2], Michael Chi-Wai Chan[3], Joseph Sriyal Malik Peiris[3], Ricky Ngok-Shun Wong[1,2,*]

Supplementary figure 1A

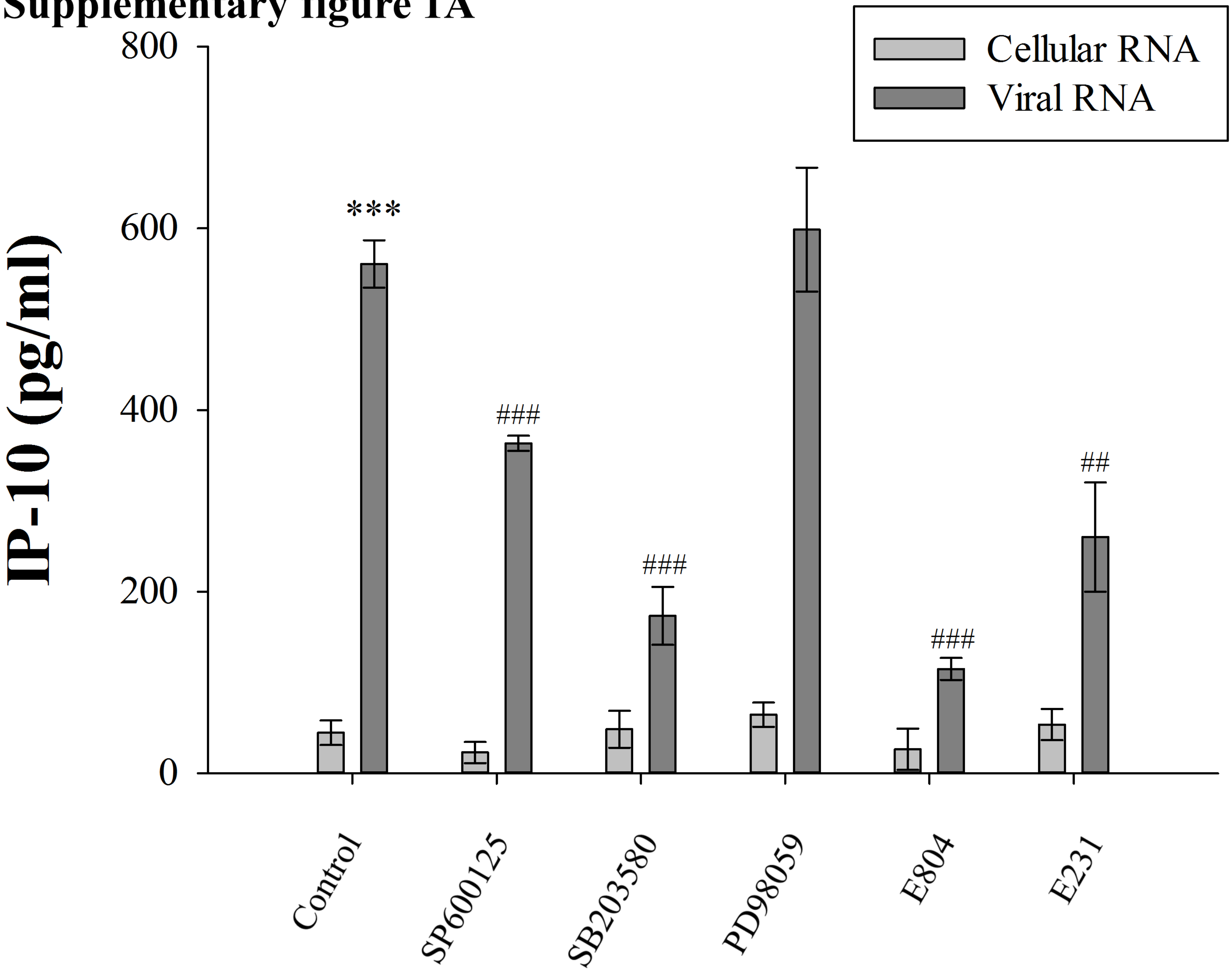

Supplementary figure 1B

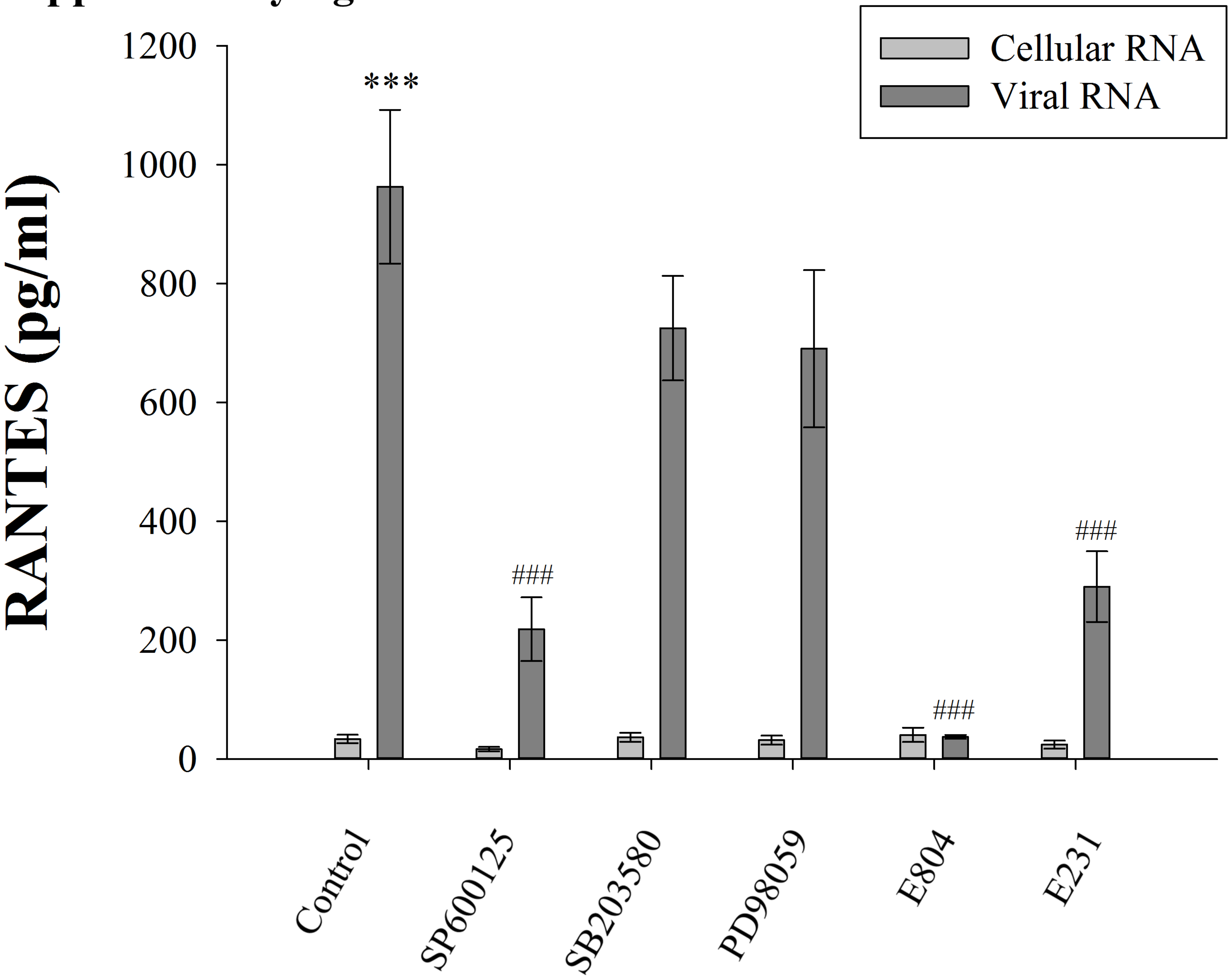

Supplementary figure 1C

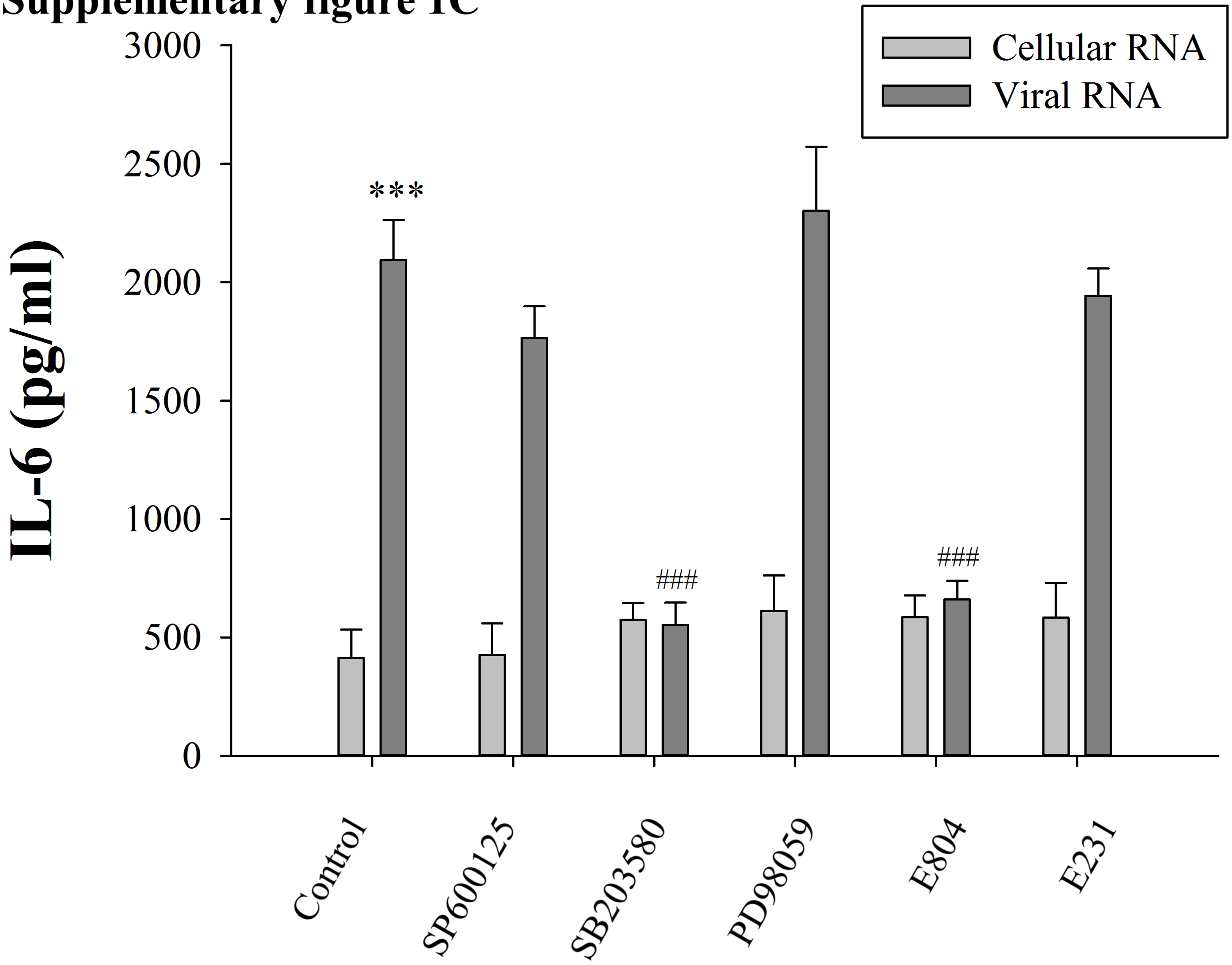

Supplementary figure 1. Indirubin derivatives and MAPKs inhibitors inhibited cytokines expressions are independent on viral load. HPMECs transfected with cellular RNA (100 ng/96-well) or viral RNA extracted from H9N2 (100 ng/96-well) were treated with either SP600125 (JNK inhibitors) (10 µM), SB203580 (p38 inhibitor) (10 µM), PD98059 (ERK inhibitor) (10 µM), indirubin derivatives E804 (1 µM) or E231 (1 µM) for 24 h.p.i.. Cell culture supernatant was harvested and the levels of (A) IP-10, (B) RANTES and (C) IL-6 were measured by ELISA. The values are presented as mean ± S.D. from three independent experiments. *** $p < 0.001$ vs mock-infected cells. ## $p < 0.01$, ### $p < 0.001$ vs cells only transfected with viral RNA.
